# Diversity in elite athlete mental health and the potential to thrive

**DOI:** 10.3389/fspor.2026.1804931

**Published:** 2026-07-29

**Authors:** Yufeng Li, Robert Joel Schinke, Yasutaka Ojio, Ale Quartiroli, Kazutoshi Kudo

**Affiliations:** 1 College of Physical Education and Health, East China Normal University, Shanghai, China; 2 School of Kinesiology and Health Sciences, Laurentian University, Greater Sudbury, ON, Canada; 3 School of Psychology, Beijing Sport University, Beijing, China; 4 Graduate School of Arts and Sciences, University of Tokyo, Tokyo, Japan; 5 Department of Psychology, University of Wisconsin-La Crosse, La Crosse, WI, United States; 6 School of Psychology, Sport, and Health Sciences, University of Portsmouth, Portsmouth, United Kingdom

**Keywords:** cultural pluralism, diversity, elite athletes, mental health, thriving

## Introduction

There has been a sizable number of standalone contributions and special issues devoted to elite athlete mental health (AMH). Until 10 years ago, the literature pertaining to elite AMH often targeted catalysts to a compromised status, primarily focusing on individual psychological factors, such as athletic identity and athletic functioning (1, 2). Some of the earliest discussions pertained to athlete retirement, with recognition that ineffective transitioning pathways contributed to personal and professional crises (38, 39). Should readers examine historically popular sport psychology textbooks, the subject areas closest to the front were mental skills training and motivational theories and processes (3, 4). There is recognition that motivational behaviors can be viewed as an output from fundamental skillsets, including holistic mental health, development, context, and staff members' professional conduct (5).

We argue that the next step in elite AMH scholarship is not to replace one dominant lens with another. Rather, it is to integrate multiple lenses in a way that better reflects athletes' lived realities. Psychopathology remains necessary for clinical precision and professional care, but it is insufficient as the only organizing framework. Thriving is important, but it cannot be reduced to positive language, nor performance success. Proactive education is essential, and it must be embedded across the athletic career and the sport system. Culture is central, but it, too, should not be regarded as a final add-on after universal models have already been defined. The gap we address is therefore conceptual and practical in keeping with our roles as scientist—practitioners (6). AMH research and service delivery can remain fragmented when clinical care, wellbeing promotion, career-long education, and cultural context are regarded as separate agendas. We propose a culturally inclusive, holistic, thriving-oriented framework in which these elements are understood as interdependent. This opinion article advances the position that elite AMH should be conceptualized as an ecological and culturally situated process that supports clinical safety, adaptive functioning, belonging, meaning, and the potential to thrive across sport and life (5, 7, 8).

This position also reflects the composition and values of the author team. The contributing authors represent four countries across North America and Asia, and our collective perspective is shaped by Western, Eastern, and cross-cultural scholarly traditions, offering a cohesive international perspective. Accordingly, our aim is not to offer critique as opposition or dismissal. Instead, we use constructive critical reflection to clarify the limits of single-lens approaches and to offer a more plural, integrative, and practice-relevant way forward. The manuscript is organized around a four-pillar framework. We (1) consider clinical precision without clinical reductionism, (2) examine thriving as a positive and trainable capacity, (3) discuss career-long proactive education as a system responsibility, and (4) position cultural pluralism and situated inquiry as central to ethical and effective AMH practice. Together, these pillars move the article beyond a descriptive review and toward an opinion that invites AMH researchers and practitioners to build knowledge through multiple, complementary perspectives.

### A culturally inclusive thriving framework for elite athlete mental health

**Figure 1:**
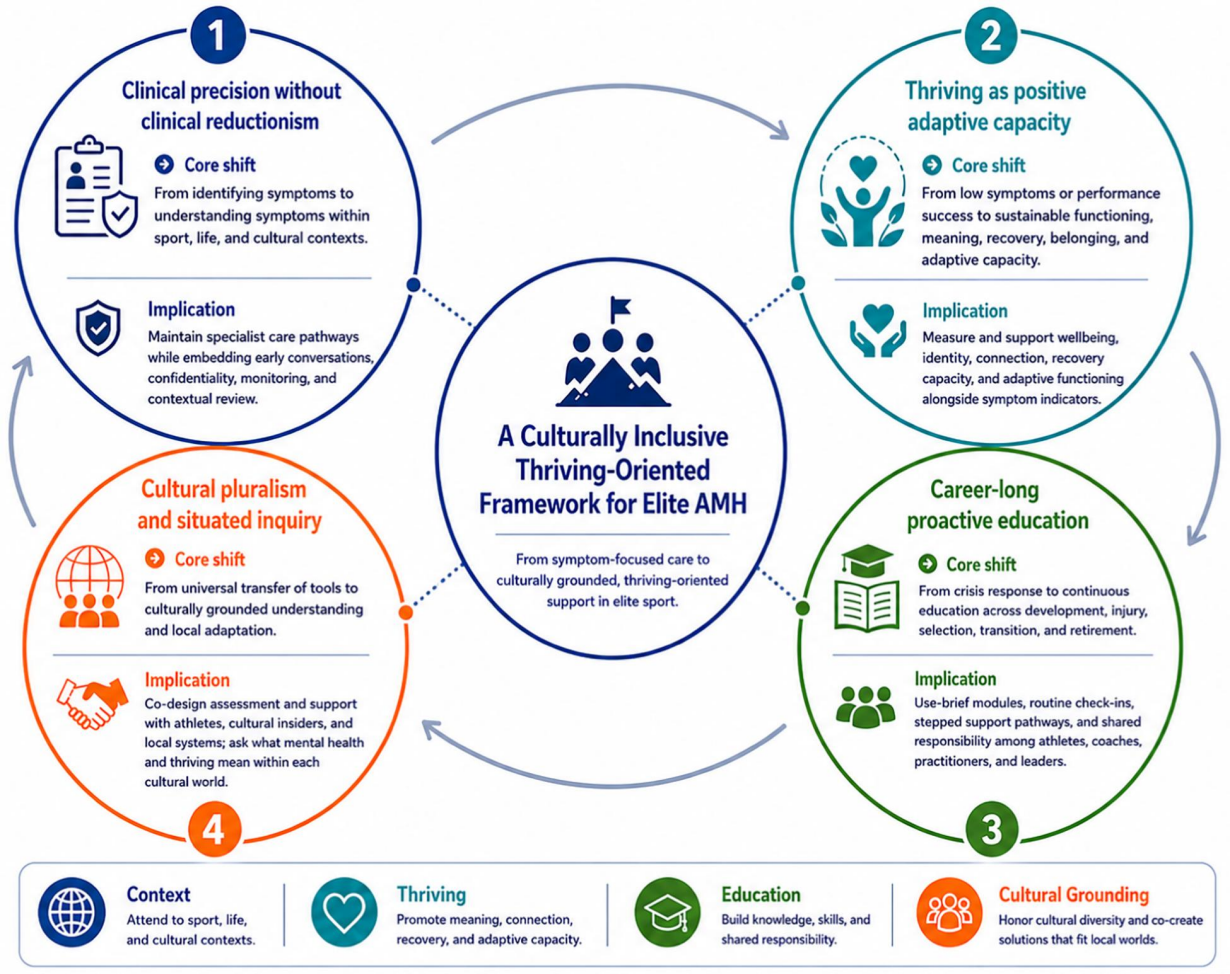
A culturally inclusive thriving-oriented framework for elite AMH.

We propose a culturally inclusive thriving framework for elite AMH (see Figure 1). The framework is not intended as a new diagnostic taxonomy or a universal intervention manual. It is a set of organizing principles for research, education, and applied practice. Its central claim is that elite AMH is best understood when clinical, developmental, environmental, and cultural processes are examined together.

The framework rests on four connected shifts: (1) from clinical precision alone to clinical precision without clinical reductionism, (2) from symptom management alone to thriving as sustainable adaptive functioning, (3) from crisis response to career-long proactive education and system responsibility, and (4) from universal transfer of tools to cultural pluralism and situated inquiry. These shifts are complementary. They allow elite AMH scholarship to retain the strengths of existing clinical and performance models while expanding the field toward more inclusive understandings of what mental health means in different sport and cultural contexts.

### Clinical precision without clinical reductionism

Psychopathology has been essential in bringing AMH into mainstream practice. It has clarified clinically significant presentations, strengthened referral pathways, and helped sport organizations respond when elite athletes experience distress (9, 10). We do not argue against psychopathology, but we argue against clinical reductionism, by which we mean the tendency to treat symptoms or diagnoses as the full story of an athlete's mental health.

Contemporary work in psychopathology and psychiatry increasingly challenge models that locate mental disorder(s) solely within the individual. Mental health and illness emerge through dynamic relations between people and their environments, with psychopathology embedded in the social, cultural, and institutional contexts shaping lived experience, distress, and recovery (40). This insight matters in elite sport because athletes' experiences are shaped by selection pressure, injury, travel, organizational hierarchies, coaching relationships, and national sport cultures. If these conditions are not considered, symptom identification can become disconnected from the lived context in which symptoms appear and are maintained.

A dual-continua view is useful because it recognizes that mental illness and wellbeing are related but distinct. Low symptoms do not necessarily indicate human thriving, and the presence of symptoms does not erase an athlete's capacity for meaning, connection, or adaptive functioning (11, 12). For this reason, athletes should pursue risk management and wellbeing promotion in parallel. High-performance systems that aim to “win well” should define success as ambitious performance pursued alongside multidimensional wellbeing across mental, physical, social, and career domains (9, 13).

We contend that symptom-focused approaches are most effective when placed within a wider system of relational and contextual care. In elite sport, impairments can remain hidden when excessive strain is normalised or when athletes learn that toughness requires silence (7). Screening alone rarely improves outcomes unless it is connected to trusted conversations, confidentiality, proportionate support options, and timely referrals, supported by regular reviews that monitor athletes' symptoms in relation to their daily contexts (9, 14). Clinical precision is necessary, but it should be held within an ecological approach that connects performance-oriented mental skills training, proactive education, and specialist clinical care.

### From psychopathology to elite athlete thriving

AMH in high-performance sport has been framed through psychopathology, prioritizing the identification, diagnosis, and treatment of psychological symptoms and disorders (10). This framing has created important gains in recognition and care. However, when used alone, it can unintentionally make mental health appear relevant only when athletes are unwell. We argue that AMH should also be understood as a positive capacity that supports performance, learning, development, and life. Mental health is dynamic and context-sensitive, fluctuating across training demands, competitive pressures, injuries, career transitions, and sociocultural conditions (2, 9). This shift moves the field beyond binary distinctions of “healthy” from “unhealthy” toward a continuum-based model, recognizing that athletes can simultaneously experience strain, adaptation, growth, and flourishing over time (15, 41). As mentioned above, the absence of mental ill health symptoms does not necessarily equate to thriving (11, 12).

Thriving can be conceptualized as sustainable adaptive functioning, recovery capacity, and resource development across physical, psychological, social, and performance domains (16, 17). Thriving includes emotional regulation, motivation, identity coherence, confidence, social connectedness, meaning, and belonging within sport environments (17). “Thriving” is not defined solely by symptom reduction; it involves maintaining valued roles, relationships, and purpose even when symptoms or stress fluctuate. Treating thriving as a core AMH objective changes the questions researchers and practitioners ask. Instead of asking only whether athletes are symptomatic, we must also ask whether they have recovery opportunities, trusted relationships, room for identity development, culturally meaningful support, and a sense that excellence can be pursued without losing health or humanity. Mental health then becomes an enabler of elite functioning, supporting resilience, adaptability, learning, and sustained engagement rather than serving only as protection against disorder.

Thriving-oriented support becomes feasible when wellbeing is treated as a trainable and monitorable set of capacities and routines. This support can be implemented through short and repeated modules. By modules, we mean brief, stand-alone AMH learning and support units, such as 30 min psychoeducational sessions, guided reflection exercises, or digital resources that can be aligned with training cycles, injury rehabilitation, selection periods, major competitions, and transitions (18–20). Access to such modules should be provided by the sport organization (e.g., clubs, national governing bodies, high-performance institutes, university programs, or professional teams), with content designed and delivered in collaboration with qualified sport psychology practitioners, mental health clinicians when appropriate, and trained athlete-support staff. Performance-focused mental skills remain valuable, but they do not fully address sleep and recovery, coping under strain, help-seeking readiness, life balance, relational safety, or cultural meanings of distress. Elite sport contexts often overlook proactive mental health support and care beyond clinical treatment, routine education that builds mental health literacy, normalizes brief routine check-ins (e.g., short self-report plus a follow-up conversation with a trusted staff member), and provides clear signposting to stepped support as needed. AMH support must extend beyond the athlete to the ecosystem, shaping affordances for recovery and connection across the training environment (21, 40).

### Career-long proactive education and system responsibility

Mental health initiatives in sport have been reactive, introduced in response to crises, performance downturns, injuries, and the emergence of clinically significant distress (10). While such responses remain necessary, reliance on reactive models risks delayed interventions, reinforcing stigma, and framing mental health as a problem to be managed rather than a capacity to be cultivated (22). We propose that proactive mental health education should be regarded as a core and continuous component of athlete development across the sporting lifespan, from early participation through retirement (8).

Rather than treating psychological support as episodic or remedial, being proactive necessitates a systematic cultivation of competencies that sustain adaptive functioning, including stress regulation, emotional awareness, motivation, identity formation, self-regulation, interpersonal effectiveness, recovery practices, and career planning (17). These competencies should be revisited across developmental phases, including entry into elite pathways, relocation, injury, deselection, major competition, transition between teams, and retirement (8).

In practice, proactive AMH education can include brief pre-season literacy modules, short self-reflection tools, confidential check-ins with trusted staff, clear stepped-care pathways, and routine conversations about sleep, recovery, relationships, identity, and life outside sport. It can also include transition planning for athletes moving from youth to senior teams, injury-specific psychological education during rehabilitation, and retirement literacy before athletes leave elite sport. These examples are not intended as a universal template. They show how proactive education can be embedded into everyday sport structures rather than reserved for moments of crisis.

Promoting AMH requires shared responsibility among practitioners, coaches, leaders, and support staff, families, and athletes themselves (23, 24). Coaches and leaders do not need to become clinicians, but they should understand how their communication, selection practices, scheduling, and responses to distress shape the psychological climate. Practitioners can support this process by training staff to recognize early signs of strain, respond without stigma, protect confidentiality, and refer athletes to appropriate care when needed (20, 25, 26).

Proactive mental health systems are further strengthened through routine monitoring, early identification practices, and structured support pathways that position mental health as a standard component of athlete care (27). Effective mental health promotion requires recognition that elite athletes' experiences of well-being, stress, and functioning are subjective, context-dependent, and shaped by performance and life demands and by their own cultural backgrounds (7). Education and support strategies must remain flexible, responsive, and collaboratively informed rather than fixed and offered as one-size-fits-all (18–20, 23). When proactive education is embedded across the career and the sport system, AMH becomes a foundational capacity that can be built, practiced, and sustained, enhancing resilience, autonomy, adaptability, and long-term psychological well-being (10, 27).

### Cultural pluralism in elite AMH and thriving

As elite AMH continues to receive global attention, it is important that we move beyond Western(ized) frameworks onward to exploring how mental health is understood, expressed, and addressed in diverse cultural contexts, with diverse clients (5). We argue that culture is not a contextual detail added after mental health has been defined. Culture shapes what distress means, how it is expressed, who is trusted, which forms of support are acceptable, and what thriving looks like in sport and life (5, 28).

Culture and cultural identity remain a powerful and underexamined force shaping how these processes unfold in real-world systems. The ways athletes experience, interpret, and respond to mental health challenges are deeply shaped by their cultural upbringing and social surroundings (2, 6, 9, 28–30). Athletes are raised in societies with unique beliefs about emotion, performance, success, and personal responsibility, and these play a key role in shaping how they express distress and seek support (5, 28). Many Western countries such as Canada or Australia, athletes are encouraged to speak openly about their mental health struggles (18, 19, 22, 27). Mental health in these settings is often viewed as an individual concern and help-seeking is regarded as a sign of inner strength (10, 18, 19). Interventions commonly emphasize personal coping strategies, mindfulness, or one-on-one therapy sessions (5).

In China, Japan, and South Korea, cultural norms rooted in Confucian values promote emotional restraint, social harmony, and respect for authority (5, 9, 31, 32, 42). Within these Eastern contexts, athletes may avoid expressing emotional difficulty, especially in public, as doing so could be seen as burdening others or bringing shame to their group given that collective priorities supersede individual health status (31). These differences signal a need for context-driven AMH models (5). The assumption that universal tools, assessments, or interventions will translate across borders risks misunderstanding athlete experiences and misapplying solutions. Using a direct questioning style in therapy or mental health assessments may lead to underreporting among athletes from cultures where emotional openness is not normative.

A commitment to cultural humility and pluralism must guide training and service delivery within international sport systems (2, 6, 7). Practitioners should adapt interventions not only to the local cultural practices, languages, and social expectations of the host setting, but also to athletes' own cultural backgrounds, migration histories, identities, and preferred ways of expressing distress and seeking support (5, 33, 34). For example, when an athlete from an Eastern cultural context relocates to a Western sport system, culturally responsive practice may require support to be tailored to the athlete's cultural worldview rather than simply to the dominant norms of the host setting. Practitioners should also collaborate with cultural insiders or trusted stakeholders who understand the athletes' worldviews and can bridge global knowledge with local and athlete-specific experiences (2, 5–7). In this sense, cultural pluralism does not weaken scientific knowledge. It strengthens science by requiring concepts, methods, and practices to remain responsive to the people and settings they aim to serve.

### Towards culturally inclusive modes of inquiry

Having established the need for culturally attuned understandings of elite AMH (2, 5), we call for a deeper inquiry into how culture shapes the psychological lives of elite athletes in performance systems. We advocate for engaging with culture as an active, structuring force that shapes how aspiring athletes perceive mental health, navigate transitions, and pursue thriving.

Future AMH inquiry should ask not only whether existing frameworks apply across cultures, but what mental health means within a cultural world (9, 28). This shift changes both research questions and methods. Researchers may need to use participatory, qualitative, mixed-methods, and culturally grounded approaches that allow athletes, coaches, families, practitioners, and local communities to define relevant meanings and outcomes. Such approaches are especially important when studying populations whose experiences have been interpreted through models developed elsewhere.

In East Asian contexts, such as China and Japan, Confucian-rooted values emphasize duty, hierarchy, and emotional control (31–33, 35). In Indigenous sporting systems in North America, Australia, and New Zealand, mental wellbeing is seen as relational and spiritual, shaped by land, ancestry, and community bonds (36). In Latin America, athletes navigate identity through shared histories of resilience, faith, and social connectedness experiences shaped by colonial legacies (28, 37). These examples demonstrate that AMH cannot be reduced to a single psychological vocabulary.

Culturally inclusive inquiry also has practical implications. Service models should be co-designed with athletes and local stakeholders. Assessment should allow indirect and relational forms of expression when direct disclosure is culturally difficult. Practitioner training should include cultural humility, reflexivity, and awareness of power relations in high-performance systems. Evaluation should track not only symptom change, but also trust, belonging, psychological safety, recovery access, help-seeking pathways, and culturally meaningful indicators of thriving.

In our view, the field advances when it welcomes multiple cultural grammars of mental health. Asking what mental health and thriving mean in a cultural world opens broader pathways for ethical, relevant, and impactful AMH knowledge. It also aligns with a pluralistic philosophy of science in which different traditions contribute together to shared understanding and practice.

## Conclusions

Elite AMH is embedded within cultural, relational, and environmental contexts and cannot be fully understood through psychopathology alone. Athletes' psychological functioning emerges from interactions among personal experiences, performance environments, social systems, and cultural conditions. Our central argument is that the field should move toward a culturally inclusive, holistic, thriving-oriented framework that integrates clinical precision, wellbeing promotion, career-long proactive education, and cultural pluralism. This argument does not dismiss existing AMH models. It places their strengths within a wider ecological and culturally situated understanding of athlete life.

The future of AMH research and practice should therefore be integrative rather than oppositional. We invite AMH researchers and practitioners to build the field through multiple perspectives. Future researchers should develop culturally grounded and co-designed service models, evaluate proactive education across the athletic career, and identify indicators of thriving that are meaningful across diverse sport systems. In this way, AMH can become a science and practice of protection, growth, belonging, and shared human development. The aim is not only to help athletes avoid disorder, but also to support their potential to thrive in sport and life.
